# Public Hygiene Campaign in Denmark during the 2009 H1N1 Pandemic Had No Effect on Hospitalization Rate of Communicable Diseases in Children

**DOI:** 10.1371/journal.pone.0070946

**Published:** 2013-07-30

**Authors:** Nadja Hawwa Vissing, Astrid Sevelsted, Hans Bisgaard

**Affiliations:** COPSAC, The Copenhagen Prospective Studies on Asthma in Childhood, Copenhagen University Hospital, Gentofte, Denmark; University of Liverpool, United Kingdom

## Abstract

**Background:**

During the 2009 H1N1 pandemic the Danish National board of Health carried out massive public hygiene campaigns to limit spread of disease. We aimed to investigate whether this resulted in lower incidences of communicable diseases in the paediatric population.

**Methods:**

The study compared annual hospitalization rates for childhood infections from 2005 to 2011.

**Results:**

Admission rates for infections were higher during the year of the pandemic compared to the rest of the period.

**Conclusion:**

There were no indications of a preventive effect by the hygiene campaign on incidence of severe common childhood infections.

## Introduction

Personal and environmental hygiene is clearly associated to spread of communicable diseases[1], and improved hand hygiene reduces transmission of infections in hospitals[2] as well as in the community[3], [4], particularly in the paediatric population [5]–[7].

In June 2009 the World Health Organization (WHO) declared a global influenza pandemic and the Danish National Board of Health responded by implementing a national pandemic action plan [8]. The first community acquired cases were seen in Denmark in October 2009 followed by rapidly increasing numbers with a peak incidence in mid-November. The pandemic began to tamper off in December with only few cases hereafter [9]–[11].

The public awareness campaign was more massive than seen in decades because of the fear for the novel and potentially deadly pandemic influenza. Public education focused on awareness on the mode of transmission of respiratory virus encouraging hand washing, covering nose and mouth when sneezing or coughing, minimizing physical contact with infected people and staying home when feeling unwell. Alcohol-based hand cleaners were widely distributed in public institutions, day care centres, schools etc. Campaigns were communicated through TV and radio spots, newspapers, public websites and distribution of written materials and posters [12]–[15]. The campaign was considered successful [14] and the National Board of Health was awarded with the annual communication price from the Danish Association for Communication Professional [16].

We hypothesized, that such increased public awareness of hygiene as prevention for influenza infection could potentially cause a lower hospital admission rate for other common communicable infectious diseases in children. Young children represent the largest burden of hospitalizations for infectious diseases. We therefore analysed the hospital admission rates in Denmark for infectious diseases in children less than six years of age during the period 2005–2010.

## Methods

Hospital admission for children aged less than six years from July 2005 to June 2011 was obtained from the National Patient Register. This register contains individual data on all hospital contacts linked with a personal identification number, dates of admission and discharge, and diagnoses at discharge classified according to the international classification of diseases (ICD-10) [17].

Cases of infections were identified as hospitalization (in-patient admission, out-patient admission, emergency room patient) where the primary diagnosis was an infectious disease. Four groups of infections were chosen and categorized as upper airway infections (ICD-10: A37, J00–J06); lower airway infections (ICD-10: J12–J22); influenza/viral infection (ICD-10: J09–J11, B09), and gastrointestinal infections (ICD-10: A02, A04, A08, A09).

Hospitalizations for the same type of infection category in the same child admitted less than 7 days apart were excluded from analysis.

Children younger than six years of age were followed for hospitalizations from July 1st 2005, or from date of birth if this occurs later, until June 31st 2011. Children who migrated or died during this period were only followed till the day of this event. The study years were divided in 12 months from July-June.

### Ethics

The study was based on data from national registries and was approved by the Danish Data Protection Agency (J.no. 2012-41-0388). Subjects were not contacted as a part of the study; hence the ethics committee did not require written informed consent.

### Statistical analysis

For each disease category we accumulated person-time and number of hospitalizations in one month intervals ranging from July 2005 to June 2012 using the macro defined by Rostgaard [18].

The admission rate describes the proportion of admissions for the four types of infections. It was defined and computed as the number of monthly admissions in the four types of infections divided by the total number of children at risk.

To test any difference in the admission rates of infections in the season of the hygiene campaigns versus the other years we compared the incidence rates in quasi Poisson regression with the log transformed person-time as offset. Analyses were done using PROC GENMOD in SAS 9.3. (SAS Institute, Inc., Cary, NC).

## Results

394,170 children born 1999–2011 were observed for hospitalizations for 2,330,174 person-years.

There were a total number of 163,194 unique hospitalizations for the four groups of infections in the period July 2005-July 2011 (table 1).

**Table 1:** Number of hospitalizations in 394,170 children during 2,330,174 person-years by age.

Age (years)	Gastro-intestinal Infections	Influenza	Upper Respiratory Tract Infections	Lower Respiratory Tract Infections
0–1	13 820	634	21 972	30 434
1–2	11 462	548	16 270	22 756
2–3	4 716	300	8 242	6 942
3–4	2 384	220	5 866	3 392
4–5	1 660	206	3 892	2 108
5–6	1 336	168	2 586	1 280

The majority of admissions were caused by acute airway infections, such as laryngitis and bronchitis. Children below three years of age accounted for the majority of admissions (85%) (table 1).

**Figure 1:**
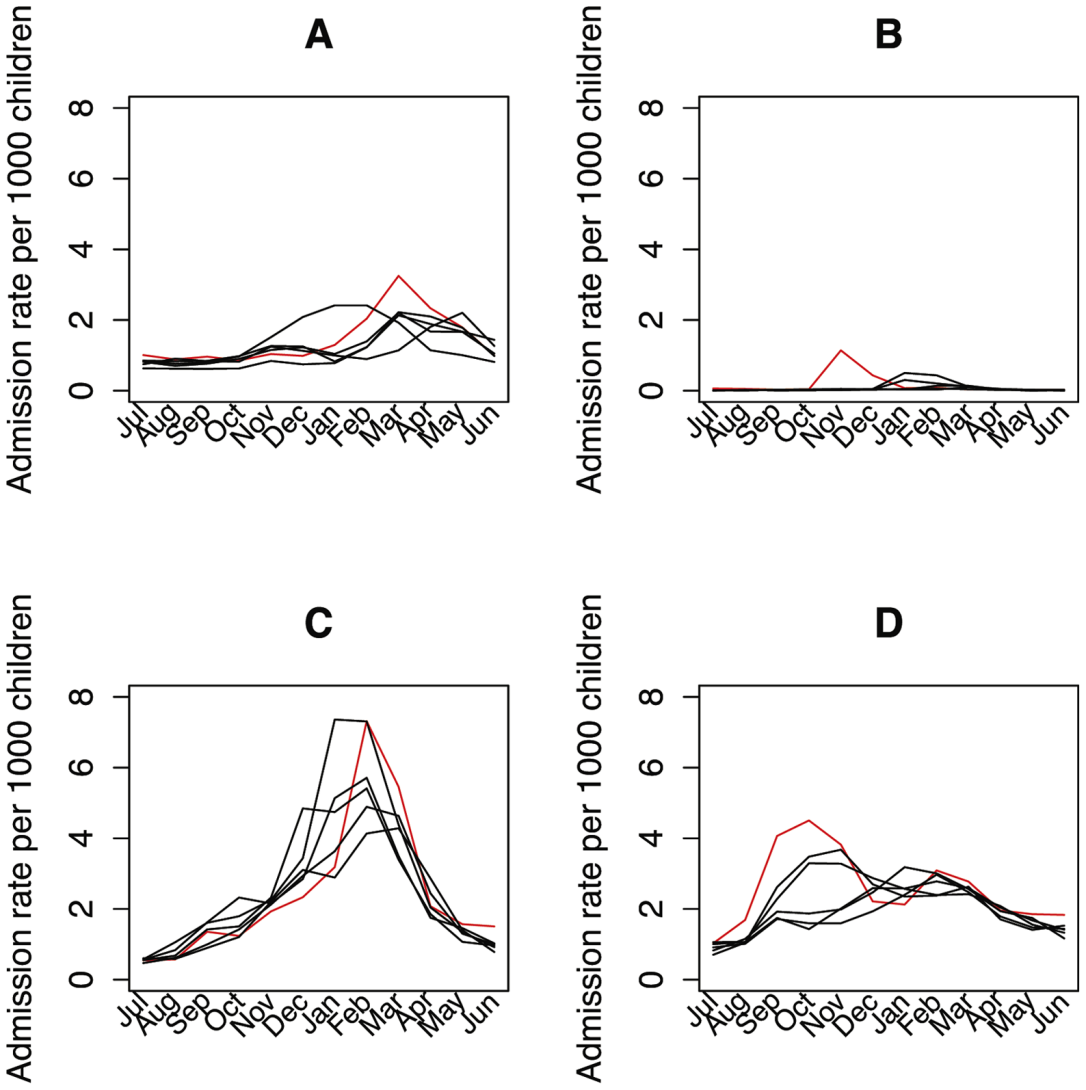
Monthly admission rates 2005–2011 by year. **Season 2009–2010 highlighted.** A: Gastroenteritis. B: Influenza. C: Lower airway infections. D: Upper airway infections.

Table 2 shows the incidence rate ratios of the H1N1 season (2009–2010) compared to the other study years. There were significantly more admissions for influenza and upper airway infections during the year of the hygiene campaigns compared to the other years. Admission rates for gastrointestinal infections and lower respiratory tract infections were not significantly different. Stratification of analysis by age groups showed similar results in infants (less than three years of age) and preschool children (three to five years of age). Figure 1 depicts the monthly admission rates, with the year 2009–2010 highlighted in red.

**Table 2:** Incidence rate ratios of admission rates, season 2009–2010 versus other seasons.

	Gastroenteritis	Lower airway infections	Upper airway infections	Influenza
*Overall*				
IRR 2009–2010 vs. others.	1.18	1.01	1.28	3.08
Age 0–5	[0.92–1.51]	[0.66–1.57]	[1.03–1.59]	[1.64–5.77]
	p = 0.1818	p = 0.9473	p = 0.0237	p = 0.0005
*By age group*				
IRR 2009–2010 vs. others.	1.23	1.00	1.27	3.08
Age 0–2	[1.03–1.47]	[0.78–1.29]	[1.08–1.49]	[1.29–7.33]
	p = 0.0196	p = 0.9985	p = 0.0034	p = 0.0111
IRR 2009–2010 vs. others.	0.93	1.16	1.34	3.07
Age 3–5	[0.66–1.30]	[0.80–1.66]	[1.05–1.72]	[1.66–5.71]
	p = 0.6543	p = 0.4366	p = 0.0184	p = 0.0004

## Discussion

We did not find lower incidence rates of paediatric hospitalizations for infectious diseases during the H1N1 pandemic 2009–2010. Any potential spill-over effect of the hygiene campaign on general paediatric health did not affect the hospitalization rates this year compared to surrounding years. In fact there seemed to be an increased rate of hospitalizations for infections in the year of the pandemic. This apparent increase may suggest a general alertness among health care workers and parents to the potential serious H1N1 infection, even spilling over to other infections. The campaign might have limited the spread of H1N1 at the peak of the pandemic, but there is no evidence pointing towards a wider effect of the intervention.

The strength in our study lies in the National Patient Register as primary data source, which is considered unique and of very high quality [19]. This registry is compulsory and links the government reimbursement to the public hospitals leading to a complete coverage. Currently public hospital healthcare is the sole option for hospitalization of children, with no private alternative available.

The interpretation of our results is limited by the fact that we only look at hospital referrals. Although this could be considered a reliable proxy for the disease burden in the community, it remains unknown whether the public campaign may have had an effect on infectious diseases in the community, i.e. on milder infections.

Since the campaign covered the entire country we are not able to compare regions with and without the campaign. Instead we compared the year of the pandemic (and the campaign) to the surrounding years. We used June-July as cut points for calendar year since the peak of infections during winter varies by year.

In conclusion, this national registry based study of hospitalization rate in Denmark was unable to detect any nationwide effect from the massive public campaign promoting good public hygiene during the 2009 H1N1 pandemic when compared to surrounding seasons where we expect less focus on hygiene.
